# Regulation of the S-Locus Receptor Kinase and Self-Incompatibility in *Arabidopsis thaliana*

**DOI:** 10.1534/g3.112.004879

**Published:** 2013-02-01

**Authors:** Susan R. Strickler, Titima Tantikanjana, June B. Nasrallah

**Affiliations:** Department of Plant Biology, Cornell University, Ithaca, New York 14853

**Keywords:** self-incompatibility, *S*-locus receptor kinase, NRPD1a, *Arabidopsis thaliana*

## Abstract

Intraspecific mate selectivity often is enforced by self-incompatibility (SI), a barrier to self-pollination that inhibits productive pollen-pistil interactions. In the Brassicaceae, SI specificity is determined by two highly-polymorphic proteins: the stigmatic S-locus receptor kinase (SRK) and its pollen coat-localized ligand, the S-locus cysteine-rich protein (SCR). *Arabidopsis thaliana* is self fertile, but several of its accessions can be made to express SI, albeit to various degrees, by transformation with functional *SRK-SCR* gene pairs isolated from its close self-incompatible relative, *Arabidopsis lyrata*. Here, we use a newly identified induced mutation that suppresses the SI phenotype in stigmas of *SRK-SCR* transformants of the Col-0 accession to investigate the regulation of SI and the *SRK* transgene. This mutation disrupts *NRPD1a*, a gene that encodes a plant-specific nuclear RNA polymerase required for genomic methylation and production of some types of silencing RNAs. We show that NRPD1a, along with the RNA-dependent RNA polymerase RDR2, is required for SI in some *A. thaliana* accessions. We also show that Col-0 *nrpd1a* mutants exhibit decreased accumulation of *SRK* transcripts in stigmas, which is not, however, responsible for loss of SI in these plants. Together, our analysis of the *nrpd1a* mutation and of *SRK* promoter activity in various accessions reveals that the *SRK* transgene is subject to several levels of regulation, which vary substantially by tissue type and by accession. This study thus helps explain the well-documented differences in expression of SI exhibited by *SRK-SCR* transformants of different *A. thaliana* accessions.

In flowering plants, out-crossing often is enforced by genetic self-incompatibility (SI), a barrier to self-fertilization that prevents pollination of the pistil by pollen from genetically related plants while allowing pollination by pollen of dissimilar genotype. In the Brassicaceae family, SI is controlled by haplotypes of the *S* locus, and self-pollination is prevented when pollen and stigma are derived from plants that express the same *S*-locus variant. Two *S*-locus genes are known to be essential for specific recognition of “self” pollen in the SI response of this family: the *S*-locus receptor kinase (*SRK*) and the *S*-locus cysteine-rich protein (*SCR*) genes (reviewed in Tantikanjana *et al.* 2010). SRK is a receptor kinase localized in the plasma membrane of stigma epidermal cells, and its SCR ligand is located in the pollen coat. An SCR variant can bind and activate only the SRK variant encoded by the same *S*-locus haplotype. This “self” SRK-SCR interaction is thought to trigger a poorly understood signal transduction cascade within the stigma epidermal cell that prevents pollen hydration and germination as well as growth of the pollen tube into the stigma epidermal cell wall (Tantikanjana *et al.* 2010).

In recent years, *Arabidopsis thaliana* has emerged as a new model system for studies of SI in the Brassicaceae (Nasrallah *et al.* 2002, 2004; Boggs *et al.* 2009; Tantikanjana *et al.* 2009). Although *A. thaliana* is normally self-fertile, it can be made to express SI by transformation with *SRK* and *SCR* gene pairs isolated from its close self-incompatible relatives *Arabidopsis lyrata* and *Capsella grandiflora* (Nasrallah *et al.* 2002; Boggs *et al.* 2009). Importantly, the SI response exhibited by *SRK-SCR* transformants of several *A. thaliana* accessions, including C24, Cvi, Kas, Hodja, and Sha, is identical in strength and developmental regulation to the SI response observed in naturally self-incompatible *A. lyrata* and other members of the Brassicaceae: it is manifested by inhibition of “self” pollen grains at the stigma surface, it is first observed in stigmas prior to flower opening (a stage that corresponds to stage 13 of flower development in *A. thaliana*), and it is sustained throughout stigma development, resulting in very low, if any, seed set (Nasrallah *et al.* 2004; Boggs *et al.* 2009). In contrast, the stigmas of *SRK-SCR* transformants of some other accessions, such as Col-0 and Rld, express transient SI: they exhibit a robust SI response only during a narrow window of stigma development (stage 13 and early stage 14), after which the ability of their stigmas to inhibit “self” pollen is weakened and they set seed (Nasrallah *et al.* 2002).

As the determinant of SI in the stigma, SRK controls the strength of SI and its proper regulation during stigma development (Tantikanjana *et al.* 2010). For example, the transient SI phenotype characteristic of some *A. thaliana* accessions is determined primarily by a hypomorphic allele of the *PLANT U-BOX8* (*PUB8*) gene, which regulates *SRK* expression: *SRK-SCR* plants homozygous for this hypomorphic *PUB8* allele accumulate suboptimal levels of *SRK* transcripts at later stages of stigma development (Liu *et al.* 2007). However, a full understanding of the SI response in the transgenic *A. thaliana* SI model and its variable expression in different accessions requires an as-yet-unavailable detailed characterization of *SRK* transgene expression and the factors essential for its proper regulation. We therefore analyzed the regulation of the *SRK* transgene and the activity of its promoter in various *A. thaliana* accessions. This study was spurred by our identification of a newly identified recessive mutation that suppresses the SI phenotype of *SRK-SCR* transformants of the Col-0 accession. The mutation disrupts *NRPD1a*, a gene that encodes a plant-specific nuclear RNA polymerase required for genomic methylation and production of some types of silencing RNAs. Here we report on our analysis of this mutation in the context of SI. We show that *NRPD1a* plays a varied and complex role in the epigenetic regulation of SI in *SRK-SCR* transformants, possibly by acting on an accession-specific factor. By analyzing other genes known to be involved in gene silencing, we identify *RDR2* as an additional requirement for the SI response in some accessions. We also show that differences among *A. thaliana* accessions for expression of SI are correlated with the activity of the *SRK* promoter.

## Materials and Methods

### Plant material and growth conditions

Transgenic plants belonging to the Col-0 accession and transformed with *SRKb* and *SCRb*, the *SRK* and *SCR* alleles derived from the *Sb* haplotype of *A. lyrata*, were previously described (Nasrallah *et al.* 2002; Tantikanjana *et al.* 2009). For plant growth, seeds were plated on MS media under sterile conditions and stratified for 3 d at 4°. Kanamycin at 25 μg/μL was used for selection of plants harboring the *SRKb* and *SCRb* transgenes. Seedlings were transplanted to soil once they had four true leaves, and plants were grown under long-day conditions (16-hr days/8-hr nights) at a temperature of 20° in a controlled-environment chamber.

### Pollination assays

Pollination tests were performed on stigmas of floral buds at stage 13 of flower development (staging according to Smyth *et al.* 1990), which corresponds to 1 d prior to flower opening and is hereafter referred to as the −1 bud stage. To identify plants exhibiting a breakdown of SI, pollination tests were performed typically by pollinating three −1 bud-stage stigmas per individual plant with pollen from a plant known to express functional SCRb, as reflected by inhibition of its pollen on the stigmas of plants expressing *SRKb*. Pollinated buds were incubated at room temperature for 2 hr and subsequently processed for visualization with a ultraviolet-fluorescence microscope as described previously (Kho and Baer 1968). Under the aforementioned conditions, very few (<5) pollen tubes are observed on the stigmas of wild-type (WT) *SRKb* plants. Plants whose stigmas exhibited more than 20 pollen tubes that penetrated the epidermal cell wall were considered to exhibit a breakdown of the SI response and were scored as self-compatible.

### Plant DNA gel blot analysis for identification of plants carrying single transgene integrations

Genomic DNA from leaf tissue was extracted using the CTAB method (Doyle and Doyle 1987) and digested overnight with *Eco*RI (New England Biolabs, Ipswich, MA). Capillary transfers were performed under alkaline conditions overnight onto Hybond N+ membrane (GE Healthcare, Bio-Sciences Corp., Piscataway, NJ). Membranes were probed with a ^32^P-labeled 1.6-kb probe derived from the 3′ UTR of the *SRKb* gene. Blots were exposed to phosphor screens (GE Healthcare Bio-Sciences Corp.) and scanned with a STORM 860 PhosphorImager (GE Healthcare, Bio-Sciences Corp.).

### Map-based cloning and DNA sequencing

A mapping population of 1600 F2 plants was generated by crossing a plant homozygous for both the *SRKb* and *SCRb* transgenes and the mutation described in this paper as female parent to a WT plant of the Landsberg *erecta* (Ler-0) accession. Plants carrying the *SRKb* transgene were identified by polymerase chain reaction (PCR) amplification of *SRKb* using genomic DNA isolated from leaves with gene-specific primers as follows: (SRKbhvrF) 5′-TGGGTTGGGATGTCAAGAAAG-3′ and (SRKbhvrR) 5′-CAACTTCATCTTTCTCAGGCACAA-3′. F2 plants carrying the *SRKb* gene were analyzed by pollinating −1-stage stigmas with pollen from a plant known to express functional SCRb (hereafter SCRb pollen). Plants showing a consistent loss of SI in these stigmas were genotyped using simple-sequence length repeats and cleaved amplified polymorphism markers listed on The Arabidopsis Information Resource website (Supporting Information, Table S1). Additional simple-sequence length repeats and single-nucleotide polymorphism markers were generated using the Landsberg *erecta* random sequence database (www.tigr.org; Table S1). A total of 125 individuals informative for mapping were found. In addition, 28 F3 plants were screened both for mapping purposes and to confirm F2 phenotypes. In this manner, the mutation was mapped to a 165-kb region on chromosome 1. To identify the gene disrupted by the mutation within the mapping interval, gene-specific primers were designed for all 39 genic regions found in the interval. PCR products were sequenced at the Cornell Bioresource Center (Ithaca, NY) using big dye terminator chemistry and AmpliTaq-FS DNA polymerase and an Applied Biosystems 3730xl DNA analyzer.

Two Col-0 SALK lines (Alonso *et al.* 2003), SALK_143437 and SALK_128428, each containing a transfer DNA (T-DNA) insertion in the *NRPD1a* gene were obtained from the Arabidopsis Biological Resource Center (ABRC, Columbus, OH). Plants homozygous for each of the T-DNA insertions were crossed to a plant homozygous for the targeted mutation and for the *SRKb* and *SCRb* transgenes for complementation assays. Additionally, the following Col-0 strains carrying T-DNA insertions in several other genes involved in silencing pathways were obtained from the ABRC (mutant designation in parentheses): SALK_059661 (*rdr2*), SALK_071772 (*ago4*), and SALK_076129 (*nrpd1b*). The C24 *nrpd1a ros1-1* double mutant was obtained from Craig Pikaard (Indiana University, Bloomington, IN). These lines were screened for homozygosity using gene-specific primers (Table S2). Plants homozygous for each of the T-DNA insertions were crossed to a Col-0 WT plant homozygous for the *SRKb* and *SCRb* transgenes, hereafter designated Col-0 WT[*SRKb*-*SCRb*] plant.

### Analysis of genomic DNA methylation by chop PCR

The DNA methylation status of plants was assayed as follows. Genomic DNA isolated from leaf tissue was digested overnight with the methylation-sensitive enzyme, *Hae*III, followed by amplification with specific primers. PCR primers for the methylated *AtSN1* repeat and for the non-methylated At2g12990 gene were as described in a previous study (Hamilton *et al.* 2002).

### RNA gel blot analysis

For RNA gel blot analysis, the following tissues were collected from 100 −1-stage flower buds: stigmas (collected by cutting pistils at the stigma-style boundary), styles (pistils lacking stigmas), petals, anthers, and sepals. PolyA RNA was extracted using the FastTrak RNA isolation kit (Invitrogen, Carlsbad, CA) and subjected to gel blot analysis as described previously (Kusaba *et al.* 2001). The blots were hybridized with a ^32^P-labeled probe generated from the first exon of *SRKb* and subsequently with a ^32^P-labeled actin probe. Visualization of hybridization signals was as described previously for DNA gel blot analysis. Signal intensities were quantified using the ImageQuant software package and normalized using actin hybridization signals.

### Reverse transcription (RT)-PCR

For quantitative real-time RT-PCR of *SRKb* transcripts, RNA was isolated from 50 stigmas or styles using the Trizol reagent (Invitrogen). Three replicate RNA samples for each genotype were prepared. For each sample, the RNA was treated with DNase I and 1 μg was used for cDNA synthesis using the First Strand cDNA Synthesis Kit for Real-time PCR (United States Biochemical, Cleveland, OH) and oligo(dT) primers. Real-time PCR was performed using iQ SYBR Green Supermix (Bio-Rad, Hercules, CA) on an ABI Prism 7900HT sequence detection system. The primers used for real-time PCR were as follows: for *SRKb* (rt-SRKb4), 5′-CTAAGCCTTGATTCTCATCTCTTTACA-3′ and 5′-GAAGTCCCCGAGCAATACCAT-3′; and for ubiquitin, gene-specific primers described in a previous study (Liu *et al.* 2007). To confirm that no genomic DNA remained in the RNA samples, both ubiquitin and *SRKb* primers were designed to span an intron. Results were analyzed using the Sequence Detection Systems software package (Applied Biosystems, Foster City, CA). The relative amounts of transcripts were calculated using the comparative ct method and normalized to ubiquitin (Livak and Schmittgen 2001). The mean values were calculated from three replicate samples collected on different days. The statistical significance of differences between genotypic groups was assessed using the Student’s *t*-test.

### Analysis of *SRKb* promoter activity using the GUS reporter

An *SRKbpr::uidA::nos* gene was constructed as follows: a fragment corresponding to a 950-base pair region upstream of the *SRKb* initiating methionine codon was amplified by PCR from an *SRKb*-containing plasmid using the forward primer 5′- ATTGTTAGTTCTTTCATCAGTTCG-3′ and the reverse primer 5′- CACTTTCCCATGGCTCTCCTTC-3′. The reporter gene was introduced into plants of the Col-0, C24, Cvi-0, Hodja, Kas, Ler-0, Rld, and Sha accessions and into Col-0 *nrpd1a*[*SRKb*-*SCRb*] plants. In addition, the previously described chimeric *Ats1pr::uidA::nos* gene (Dwyer *et al.* 1994) was introduced into Col-0 and C24 as a control stigma-specific reporter. All transformations were performed using the *Agrobacterium*-mediated floral dip method (Clough and Bent 1998). Selection was on MS medium containing 40 μg/μL hygromycin for *SRKbpr::uidA::nos* transformants and 25 ug/ul kanamycin for *Ats1pr::uidA::nos* transformants. Homozygous plants carrying a single transgene integration were identified by DNA gel blot analysis and progeny screening on selective media. Histochemical assays for GUS activity were performed as described previously (Kusaba *et al.* 2001).

To quantitate GUS transcripts, real-time RT-PCR was performed using total RNA isolated from stigmas harvested from 30 floral buds. Real-time RT-PCR was performed as described previously using the *GUS* gene-specific primers 5′-TCCTACCGTACCTCGCATTACC-3′ and 5′-GACAGCAGCAGTTTCATCAATCAC-3′. Real-time RT-PCR of ubiquitin transcripts used primers described previously (Liu *et al.* 2007).

## Results

### Identification of *NRPD1a* as a modifier of SI in Col-0[*SRKb*] plants

Col-0 plants harboring the *SRKb* and *SCRb* genes, which are the *SRK* and *SCR* alleles isolated from the *Sb* haplotype of *A. lyrata*, express a robust SI response in −1-stage floral buds (Nasrallah *et al.* 2002). To identify mutations that cause loss of SI in these plants, we used a previously described Col-0 homozygous strain carrying *SRKb* and *SCRb* genes integrated at single but unlinked positions in the genome (Nasrallah *et al.* 2002). Seeds from this strain were mutagenized with ethyl methane sulfonate, and M2 plants derived from these mutagenized plants were screened by manual self-pollination of stigmas at the −1-bud stage as previously described (see *Materials and Methods*) (Tantikanjana *et al.* 2009). This screen identified a mutation, designated *self-compatible 1* (*sc1*), which caused breakdown of SI in −1-stage floral buds of *SRKb* transformants. Pollination of *sc1* mutants carrying the *SRKb* transgene (hereafter *sc1*[*SRKb*]) stigmas with pollen from WT plants harboring the *SCRb* transgene (hereafter WT[*SCRb*]) resulted in profuse pollen tube growth, whereas pollination of WT[*SRKb*] plants with *sc1*[*SRKb*] pollen did not result in pollen tube growth, indicating that the self-compatible phenotype of *sc1*[*SRKb*] plants was due to loss of stigma SI function. Crosses between an *sc1*[*SRKb*] mutant plant and WT[*SRKb*] restored SI in the −1-stage stigmas of F1 plants, demonstrating that the *sc1* mutation is recessive. Additionally, when the *sc1*[*SRKb*] mutant was crossed to a Col-0 WT plant, the F1 plants were self-incompatible, demonstrating that the mutant phenotype is not the result of a loss-of-function mutation in the *SRKb* transgene.

Using the map-based cloning strategy described in *Materials and Methods*, we mapped the *sc1* mutation to a 165.5-kb region between markers F16P17b and F9N12c on chromosome 1 (Table S1). Sequencing of the 39 genes within this region revealed a cytosine-to-thymine transition at position 3188 in the eighth exon of the *NRPD1a* gene, which results in a glutamine-to-asparagine substitution. The *sc1*[*SRKb*] mutant was then crossed to two Col-0 SALK lines (Alonso *et al.* 2003), SALK_143437 and SALK_128428, which contain a T-DNA insertion in the *NRPD1a* gene within exon 14 and exon 9, respectively (Swarbreck *et al.* 2007). The inability of both T-DNA lines to complement the phenotype of *sc1*[*SRKb*] plants demonstrates that a defect in *NRPD1a* is responsible for the breakdown of SI in this mutant. Because seven mutations (*nrpd1a-1* to *nrpd1a-7*) had already been identified in *NRPD1a*, the *sc1* mutation was named *nrpd1a-8*.

NRPD1a is a plant-specific nuclear RNA polymerase that functions in various silencing phenomena including the generation of small RNAs (Herr *et al.* 2005), the methylation of DNA (Onodera *et al.* 2005), and the spread and reception of the silencing signal (Palusa *et al.* 2007; Scascitelli *et al.* 2010). Alignments of the predicted amino-acid sequences of NRPD1a and RNA polymerase II from yeast had identified the putative structural domains of NRPD1a (Herr *et al.* 2005). Based on these alignments, the *nrpd1a-8* mutation is located in the region homologous to the binding site of transcription factor IIb in yeast RNA polymerase II (Herr *et al.* 2005), specifically at amino acid 614 in the putative dock domain (Herr *et al.* 2005).

### *NRPD1a*, DNA methylation, and the regulation of *SRKb* gene expression

**Figure 1:**
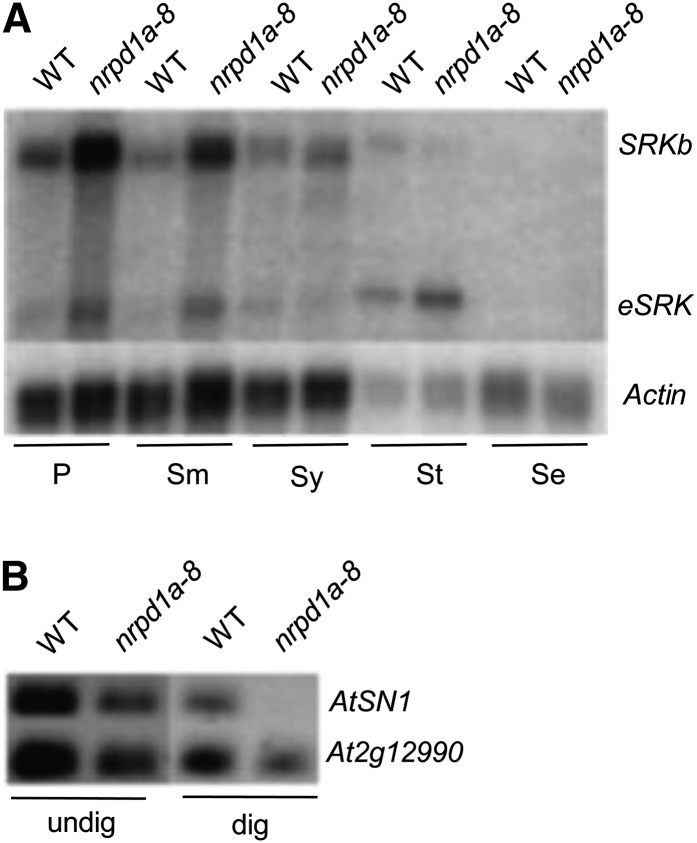
Analysis of *SRKb* expression and genomic DNA methylation in *nrpd1a-8* mutants. (A) Expression of *SRKb* and *eSRKb* in various floral tissues of *nrpd1a-8* mutants and WT. P, petal; Sm, stamen; Sy, style; St, stigma; Se, sepal. (B) Loss of genomic methylation at the *AtSN1* retroelement in *nrpd1a-8* mutants. undig, undigested; dig, digested.

Suboptimal levels of *SRK* in stigmas are known to lead to weakening or loss of SI (Liu *et al.* 2007). Consequently, although *nrpd1a* mutations are expected to relieve the silencing of transgenes and therefore to increase the expression level of the *SRKb* transgene, we explored the possibility that the self-compatible phenotype of −1-stage stigmas in *nrpd1a-8*[*SRKb*] mutant plants might be due to reduced *SRKb* transcripts. Accordingly, we compared the relative amounts of *SRKb* transcripts in *nrpd1a* mutants harboring the *SRKb* transgene (designated *nrpd1a-8*[*SRKb*]) and WT[*SRKb*] plants. As previously described and shown by the gel blots in Figure 1A, the *SRKb* gene, like other *SRK* genes, produces two transcripts: a 3.0-kb transcript that encodes the full-length SRK protein and a 1.6-kb transcript, designated *eSRK*, which corresponds to the first exon of the gene and encodes a soluble form of the SRK extracellular domain whose function is not understood (Stein *et al.* 1991; Dixit *et al.* 2000; Kusaba *et al.* 2001). Furthermore, the *SRKb* gene is expressed in various floral tissues: in addition to stigmas, *SRKb* transcripts were detected in the styles, stamens, and petals of Col-0 WT[*SRKb*] plants (Figure 1A). This pattern of expression is comparable with the native expression pattern of the *SRKb* gene in *A. lyrata* (Figure S1), with the exception of expression in stamens, which might be due to position effects of the site of transgene integration in Col-0 WT[*SRKb*] plants.

Comparison of *nrpd1a-8*[*SRKb*] and WT[*SRKb*] plants showed that the *nrpd1a-8* mutation did affect the steady-state levels of *SRKb* transcripts, albeit in unexpected ways. In stigmas, the levels of the ~3-kb *SRKb* transcript were approximately 2.2-fold lower in *nrpd1a-8*[*SRKb*] plants than in WT[*SRKb*] plants, whereas the 1.6-kb *SRKb* transcripts were approximately 2-fold more abundant in *nrpd1a-8*[*SRKb*] plants than in WT[*SRKb*] plants (Figure 1A). In contrast, both *SRKb* and *eSRKb* transcripts were elevated in the styles, petals, and stamens of *nrpd1a-8*[*SRKb*] mutants relative to WT[*SRKb*] plants (Figure 1A). The *nrpd1a-8* mutation did not, however, cause accumulation of *SRKb* transcripts in sepals, where the *SRKb* gene is not normally expressed (Figure 1A).

NRPD1a is known to function in the production of natural antisense (NAT) RNA and in DNA methylation (reviewed in Vaucheret 2006), both of which can have significant effects on gene expression. A previous study had shown that mutations in several genes of the NAT pathway did not exhibit loss of the SI response in Col-0[*SRKb*-*SCRb*] plants (Tantikanjana *et al.* 2009), indicating that *NRPD1a* does not exert its effect on SI via this pathway. We therefore assessed the possibility that NRPD1a regulates SI by regulating the methylation status, and therefore expression, of the *SRKb* gene or other genes that function in SI. We first examined *nrpd1a-8* homozygotes to determine whether they exhibit a loss of DNA methylation similar to previously analyzed *nrpd1a* mutants. Toward this end, we used a standard method that assays methylation of the highly methylated *AtSN1* retroelement. In this so-called “chop PCR” method (Onodera *et al.* 2005), genomic DNA is digested with the methylation-sensitive enzyme *Hae*III before PCR amplification with *AtSN1*-specific primers, and an amplification product is obtained only if the DNA is methylated and therefore insensitive to *Hae*III digestion. The At2g12990 gene, which contains no methylation sites, is used as a control. Figure 1B shows that *AtSN1* fragments could not be amplified from *Hae*III-digested genomic DNA of *nrpd1a-8* homozygotes, indicating that the *nrpd1a-8* mutation causes loss of genomic DNA methylation similar to other *nrpd1a* mutations.

To examine further the role of DNA methylation in the regulation of SI, strains containing T-DNA insertions in genes known to function in the DNA methylation pathway, including *NRPD1a, NRPD1b, RDR2*, and *AGO4* (Matzke *et al.* 2009), were each crossed to a WT Col-0 plant carrying a single integration of a linked *SRKb*-*SCRb* gene pair (Nasrallah *et al.* 2004). NRPD1b is an alternative large subunit of Pol IV (Pontier *et al.* 2005), whereas RDR2 amplifies RNAs cleaved by AGO4 (Matzke *et al.* 2009). Based on the chop PCR assay, plants homozygous for the T-DNA insertion in each of these genes showed the expected loss of methylation of the *AtSN1* retroelement (Figure S2). For each mutant line, F2 progenies that contained the *SRKb-SCRb* transgenes and were homozygous for the insertional mutation were analyzed by pollination assays of stigmas at the −1 bud stage. Of the mutants tested, only *rdr2*[*SRKb-SCRb*] plants exhibited a stigma-specific breakdown of SI similar to *nrpd1a*[*SRKb-SCRb*] plants. All other mutants exhibited an intense SI response in stigmas at the -1-bud stage.

**Figure 2:**
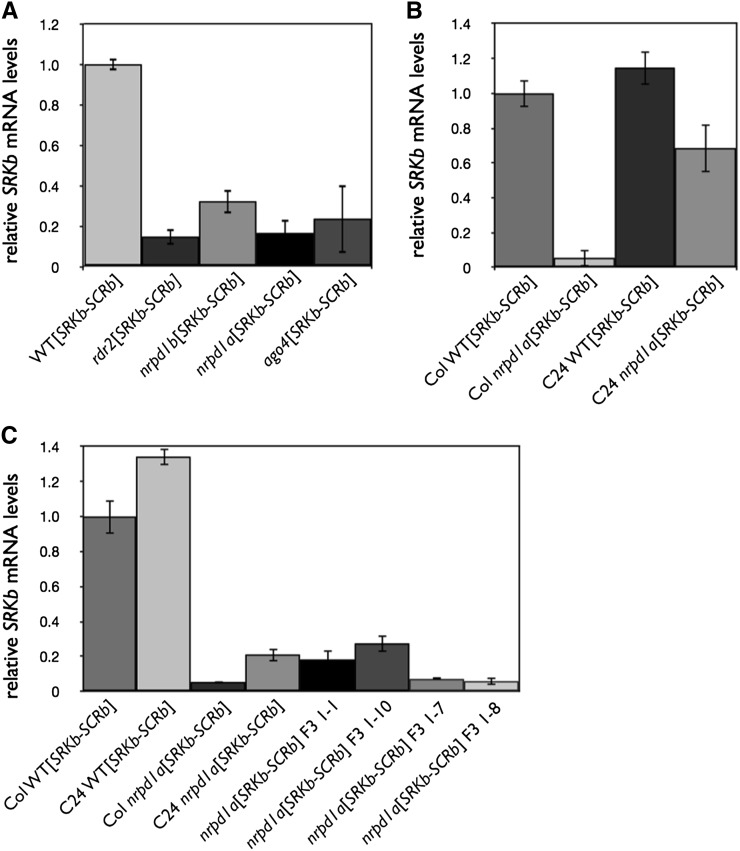
Effect of various mutations on the levels of *SRKb* transcripts in the stigmas of plants homozygous for the *SRKb-SCRb* trasngenes and for various genomic methylation mutations. Average fold-change values relative to WT[*SRKb-SCRb*] are shown for (A) *rdr2*, *nrpd1b*, *nrpd1a*, and *ago4* mutants in the Col-0 background; (B) Col-0 *nrpd1a* [*SRKb-SCRb*] and C24 *nrpd1a*[*SRKb-SCRb*]; (C) Col-0 *nrpd1a-8* × C24[*SRKb-SCRb*] F3 progeny. Among *nrpd1a*[*SRKb-SCRb*] F3 plants, plants 1−1 and 1−10 were self-incompatible whereas plants 1−7 and 1−8 were self-compatible.

The different effects on SI of the *nrpd1a* and *rdr2* mutations on the one hand and of mutations in genes of the NAT and DNA methylation pathways on the other hand might be due to differences in the effect of these mutations on *SRKb* transcript levels. To address this issue, we compared *SRKb* transcript levels in the self-compatible *nrpd1a*[*SRKb-SCRb*] and *rdr2*[*SRKb-SCRb*] plants and in the self-incompatible *nrpd1b*[*SRKb-SCRb*] and *ago4*[*SRKb-SCRb*] plants by real-time PCR using primers complementary to sequences in exons 4 and 5 and therefore specific for the full-length *SRKb* transcript. All methylation mutants tested were found to have a significant decrease in expression of full-length *SRKb* transcripts in the stigma (Figure 2A). The self-incompatible −1-bud stigmas of *nrpd1b*[*SRKb-SCRb*] and *ago4*[*SRKb-SCRb*] plants tended to have slightly greater expression than the self-compatible −1-bud stigmas of *nrpd1a*[*SRKb-SCRb*] and *rdr2*[*SRKb-SCRb*] plants. However, this difference was not significant in comparisons of *ago4*[*SRKb-SCRb*] with the self-compatible *nrpd1a*[*SRKb-SCRb*] (*P* = 0.8) and *rdr2*[*SRKb-SCRb*] plants (*P* = 0.4).

### Differential regulation of *SRKb* in different *A. thaliana* accessions

To determine whether disruption of the *NRPD1a* gene could cause breakdown of SI in *SRKb-SCRb* transformants of accessions that express a developmentally stable SI response, an *nrpd1a* mutant in the C24 background (Zheng *et al.* 2008) was obtained and crossed to a C24[*SRKb-SCRb*] plant. All 16 F2 plants that inherited the *SRKb-SCRb* transgenes were self-incompatible as demonstrated by little to no seed set. Among these plants, three *nrpd1a*[*SRKb-SCRb*] homozygotes showed a decrease in stigma *SRKb* transcript levels relative to C24 WT[*SRKb-SCRb*] (Figure 2B), despite having a self-incompatible phenotype. However, *SRKb* transcript levels in C24 *nrpd1a*[*SRKb-SCRb*] homozygotes were still ~12 fold greater than those observed in Col-0 *nrpd1a*[*SRKb-SCRb*] stigmas.

In parallel, a cross was made between a Col-0 *nrpd1a-8*[*SRKb*] homozygote and a C24 WT[*SRKb-SCRb*] homozygote, both of which contained a single integration of the *SRKb* or *SRKb-SCRb* transgenes. The F1 progeny of this cross were fully self-incompatible at all stages of stigma development. A total of 50 F2 plants from four different F1 plants were analyzed, and F2 plants were recovered whose stigmas did not inhibit *SCRb* pollen despite harboring the *SRKb* transgene. The ratios of “compatible” (C; stigmas failed to inhibit SCRb pollen) to “incompatible” (I; stigmas inhibited SCRb pollen) plants in these families were: 3 C:11 I in Family 1; 0 C:11 I in Family 2; 1 C:12 I in Family 3; and 1 C:11 I in Family 6. All of the “compatible” plants were homozygous for the *nrpd1a-8* mutation, and their F3 progenies were uniformly “compatible”. However, some F2 *nrpd1a-8* homozygotes were ”incompatible” (five of eight plants in Family 1; five of five plants in Family 2; two of three plants in Family 3; one of two plants in Family 6). All of the F2 *SRKb* plants that were homozygous or heterozygous for the *NRPD1a* WT allele were “incompatible.” Among F2 *nrpd1a-8*[*SRKb*] plants, the overall phenotypic ratio of “incompatible” to “compatible” plants was 2.6:1 (13 I:5 C). This ratio is not statistically different from the 3:1 ratio expected for segregation of a recessive allele at a locus other than *NRPD1a* that is required for SI (*P* = 0.23). Interestingly, all F2 *nrpd1a-8*[*SRKb*] plants tested, whether they were “incompatible” or “compatible,” were found to exhibit loss of *AtSN1* methylation (Table S2). Additionally, based on DNA gel blot analysis, there was no correlation among F2 plants between SI phenotype and the source of the *SRKb* transgene (*i.e.*, whether it was derived from the Col-0 or C24 parent) or the number of *SRKb* transgene integrations (*i.e.*, whether the plant contained the Col-0-derived transgene, the C24-derived transgene, or both) (Table S2). Similar results were obtained in a cross between a Col-0 *nrpd1a-8*[*SRKb*] homozygote and an *SRKb-SCRb* transformant of Sha, an accession that expresses developmentally-stable SI like C24. In this cross, some but not all *nrpd1a* F2 plants exhibited a self-compatible phenotype with a ratio of self-incompatible to self-compatible plants of 7:2 (Table S3).

To determine whether variation in *SRKb* expression may be responsible for the observed phenotypes in these F2 populations, F3 *nrpd1a* homozygotes derived by selfing F2 individuals derived from the Col-0 *nrpd1a-8*[*SRKb*] × C24 WT[*SRKb-SCRb*] cross were generated and subjected to real-time PCR using stigma RNA. Among four F3 *nrpd1a* homozygotes tested (Figure 2C), two plants exhibited an incompatibility response toward SCRb pollen in pollination tests and expressed *SRKb* at levels comparable to C24 *nrpd1a*[*SRKb-SCRb*] plants as shown through real-time PCR analysis. In contrast, the remaining two plants exhibited a compatible response toward SCRb pollen and their *SRKb* transcript levels were on average threefold lower than those in C24 *nrpd1a*[*SRKb-SCRb*] plants.

At least two modifier loci have been shown to contribute to differences in the SI phenotype of *SRKb-SCRb* transformants of different accessions: *PUB8* on chromosome 4 (Liu *et al.* 2007) and another as-yet-uncharacterized locus on chromosome 3 (Boggs *et al.* 2009). To determine whether either one of these modifier loci might be responsible for the accession-specific effect of *nrpd1a-8* on SI, markers tightly linked to these modifiers were used to analyze F2 progenies of the Col-0 *nrpd1a-8*[*SRKb*] × C24 WT[*SRKb-SCRb*] cross. However, no association was found between SI phenotype and either of these markers. Additional markers located throughout the genome were therefore tested, and loose linkage of a putative modifier locus to marker NGA139 was detected on chromosome 5 (Table S4).

### Differential activity of the *SRKb* promoter in *A. thaliana*

To understand the differential regulation of *SRKb* in the *nrpd1a* mutant and in various *A. thaliana* accessions, it is important to determine if differences in the steady-state levels of stigma *SRKb* transcripts result from differences in the transcription or stability of these transcripts. To address this issue, we assayed the activity of the *SRKbpr::uidA:nos* reporter gene in the pistils of the *nrpd1a* mutant and of several accessions that had previously been analyzed for expression of SI by transformation with the *SRKb-SCRb* gene pair (Nasrallah *et al.* 2004; Boggs *et al.* 2009): C24, Cvi, Hodja, Kas, and Sha, all of which express intense and developmentally-stable SI; and Col-0 and Rld as representatives of accessions that express *PUB8*-mediated transient SI (Liu *et al.* 2007).

**Figure 3:**
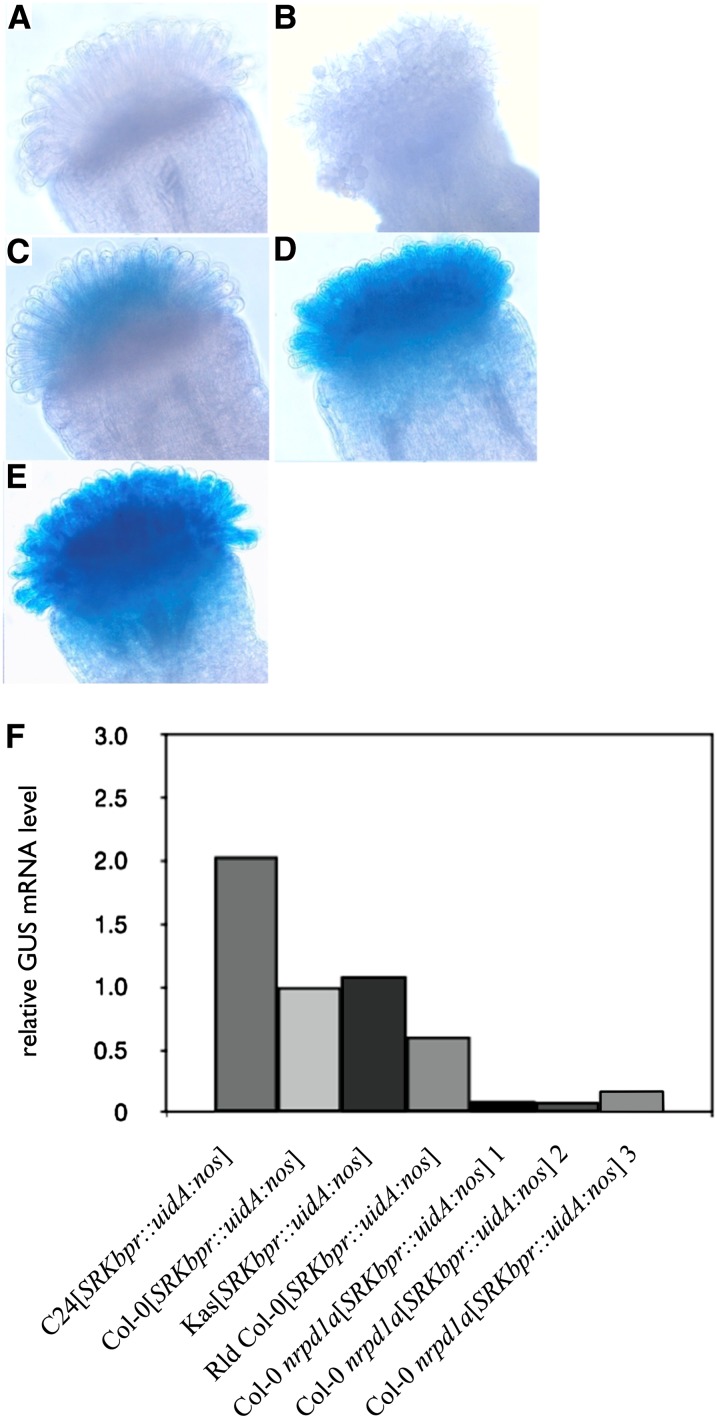
Differential activity of the *SRKb* promoter in the Col-0 *nrpd1a* mutant and in various *A. thaliana* accessions. Activity of the *SRKbpr::uidA:nos* reporter was assayed in flowers at the +1-stage of development by histochemical analysis of GUS activity and by real-time RT-PCR of stigma tissue. (A−D) *SRKbpr::uidA:nos* transformants (A) Col-0 (B) Col-0 *nrpd1a* (C) Rld, and (D) C24. (E) Col-0[*AtS1pr::uidA:nos*] used as control. (F) Real-time RT-PCR of GUS transcripts in stigma tissue of *SRKbpr::uidA:nos* transformants of four *A. thaliana* accessions and the Col-0 *nrpd1a* mutant.

In histochemical analysis of GUS activity in Col-0 *nrpd1a* plants or Col-0 WT plants transformed with the *SRKbpr::uidA:nos* reporter, no staining was observed in stigma epidermal cells or other cells of the pistil (Figure 3, A and B; Table S1), and only little staining was observed in the stigma of *Rld*[*SRKbpr::uidA:nos*] transformants (Figure 3C; Table S1). In contrast, in all accessions that express developmentally-stable SI, *SRKbpr::uidA:nos* transformants were identified which exhibited GUS activity in stigma epidermal cells, and the majority of these transformants also exhibited GUS activity in the style (Figure 3D; Table S1). Interestingly, Col-0 plants transformed with an *AtS1pr::uidA::nos* construct exhibited strong GUS staining in stigma epidermal cells (Figure 3E), as expected for the stigma epidermal cell-specific *AtS1* promoter (Dwyer *et al.* 1994). Thus, the lack of GUS staining in stigma epidermal cells of Col-0[*SRKbpr::uidA:nos*] plants is not due to general misregulation of promoters expressed in these cells, but was specific to the *SRKb* promoter.

The lack of GUS staining in the pistils of Col-0[*SRKbpr::uidA:nos*] transformants and the weak GUS staining in Rld[*SRKbpr::uidA:nos*] transformants was surprising given the fact that *SRKb*-*SCRb* transformants of these accessions express SI in −1 bud-stage stigmas and that *SRKb* transcripts are detected in Col-0 [*SRKb*] stigmas by RNA gel blot analysis (Figures 1A and 2). To investigate this discrepancy, real-time PCR was used to compare the levels of stigma GUS transcripts in strains that showed no or little GUS staining on the one hand and in strains that exhibited strong GUS staining in stigma epidermal cells on the other hand. Figure 3F shows that GUS transcripts were detected in Col-0[*SRKbpr::uidA:nos*], Rld[*SRKbpr::uidA:nos*], and Col-0 *nrpd1a* [*SRKbpr::uidA:nos*] stigmas, albeit at lower levels than in the stigmas of C24 and Kas [*SRKbpr::uidA:nos*] transformants. GUS transcript levels in Col-0 and Rld transformants were, respectively, 2.2- and 1.4-fold lower than in C24 transformants and 1.7- and 1.1-fold lower than in Kas transformants. In Col-0 *nrpd1a* [*SRKbpr::uidA:nos*] plants, GUS transcript levels were 12- to ~24-fold lower than in C24[*SRKbpr::uidA:nos*] plants. Thus, the failure to observe GUS staining in the stigmas of [*SRKbpr::uidA:nos*] transformants of Col-0, Rld, and *nrpd1a* may be attributed to the relatively low sensitivity of histochemical GUS staining.

## Discussion

The results described here reveal an unexpected complexity in the regulation of the *SRKb* transgene in *A. thaliana*. We found that *NRPD1a* is required for expression of SI in stigmas of Col-0[*SRKb*] plants. Because *NRPD1a* functions in transgene silencing at least partly by increasing DNA methylation, disruption of this gene is expected to cause reduced methylation and consequently increased expression of the *SRKb* transgene. In fact, *SRKb* plants homozygous for the *nrpd1a-8* mutant allele identified here exhibited reduced overall genomic DNA methylation as determined by loss of methylation at the *AtSN1* retroelement. They also accumulated increased levels of full-length *SRKb* and *eSRKb* transcripts relative to WT[*SRKb*] homozygotes in styles, stamens, and petals, in keeping with the abrogation of transgene silencing expected in *eSRKb* loss-of-function mutants. Unexpectedly, however, *nrpd1a-8*[*SRKb*] plants exhibited reduced steady-state levels of full-length *SRKb* transcripts relative to WT[*SRKb*] stigmas. In contrast, *eSRKb* transcripts levels were increased in *nrpd1a*[*SRKb*] stigmas relative to WT[*SRKb*] stigmas. A possible explanation for this difference in the effect of *nrpd1a* on the two *SRKb* transcripts is that the *nrpd1a* mutation causes different effects on the transcriptional machinery necessary for the production of these two transcripts.

The puzzling opposite effects of the *nrpd1a-8* mutation on *SRKb* transcript levels in stigmas on the one hand and in styles, stamens, and petals on the other hand, suggest the existence of tissue-specific factors that differentially regulate expression of the *SRKb* transgene in different floral tissues. The existence of stigma-specific factors that control *SRKb* expression would not be surprising. Indeed, stigma-specific factors that regulate *SRKb* kinase activity have been previously invoked to explain the fact that the SRKb protein exhibits SCR-dependent activation in the stigma, where it functions in recognition of self pollen, but displays constitutive SCR-independent activity in the style, where it enhances cell division (Tantikanjana *et al.* 2009). Whether *SRKb* regulatory factors common to styles, stamens, and petals exist, and whether expression of *SRK* in non-pistil floral tissues has any biological significance remain to be determined.

There is a lack of a direct correlation between genomic DNA methylation status and SI phenotype in *nrpd1a*[*SRKb-SCRb*] plants. *SRKb-SCRb* plants homozygous for the *nrpd1a, nrpd1b, ago4*, and *rdr2* mutations all showed a loss of genome methylation at the *AtSN1* retroelement, but only *nrpd1a*[*SRKb-SCRb*] and *rdr2*[*SRKb-SCRb*] plants exhibited a breakdown of SI. Additionally, in the Col-0 *nrpd1a-8*[*SRKb*] × C24 WT[*SRKb-SCRb*] cross, all *nrpd1a-8* mutant progenies exhibited a loss of genome methylation, but some retained SI. Silencing pathways in plants are known to be complex and various mechanisms exist by which DNA methylation may be initiated (Matzke *et al.* 2009; Jauvion *et al.* 2012). For example, impairment of RDR2 may reduce substrate competition between RDR2 and RDR6 and increase methylation in *rdr2* mutants (Jauvion *et al.* 2012). The use of different pathways for *SRKb* transgene silencing and for genomic methylation would explain the increased silencing of *SRKb* in the stigma in the context of the overall reduced genomic DNA methylation observed in *nrpd1a* homozygotes.

Intriguingly, there was no absolute correlation between pollination phenotype and *SRKb* transcript levels. Although *nrpd1a* and *rdr2* [*SRKb-SCRb*] plants that exhibited breakdown of SI often accumulated lower levels of *SRKb* transcripts than plants that retained SI, some plants, such as *ago4*[*SRKb-SCRb*] plants, retained SI despite expressing equally low *SRKb* transcript levels. Thus, the reduced accumulation of *SRKb* transcripts is not in itself sufficient to explain the complete breakdown of SI in *nrpd1a* or *rdr2* homozygotes. Rather, our results suggest that at least one stigma factor found in the Col-0 genetic background is responsible for the self-fertile phenotype of Col-0 *nrpd1a*[*SRKb*] plants. In particular, the fact that disruption of the *NRPD1a* gene did not abrogate the SI phenotype of C24[*SRKb-SCRb*] plants, the 3:1 ratio of self-incompatible to self-compatible plants observed among *nrpd1a-8* homozygous progenies of the Col-0 *nrpd1a-8*[*SRKb*] × C24 WT[*SRKb-SCRb*] cross, and preliminary evidence that implicates a locus on chromosome 5 in the breakdown of SI in Col-0 *nrpd1a-8*[*SRKb*] plants all suggest that NRPD1a and RDR2 act on a Col-0 locus that is involved in SI and whose phenotypic effect requires homozygosity for *nrpd1a*.

At present, we do not know what the relationship is, if any, between the factor encoded by this Col-0 locus and the accession-specific stigma factor that allows high-level expression of the *SRKb::uidA::nos* reporter in the stigmas of accessions that exhibit developmentally-stable SI but only little or no expression of the reporter in accessions that exhibit transient SI. It is clear however, that both factors act *in trans* on the *SRKb* promoter, as evidenced by the reduced GUS transcript levels observed in Col-0 *nrpd1a*[*SRKbpr::uidA::nos*] transformants.

Overall, our results are consistent with the conclusion that the *SRKb* transgene is subject to several levels of regulation that vary substantially by accession and by tissue, with involvement of epigenetic factors (as revealed by the misregulation of the *SRKb* gene in *nrpd1a* and other DNA methylation mutants) that might converge on the *SRKb* promoter. The nature of these factors and the mechanism(s) by which changes in DNA methylation effect changes in *SRKb* transcript levels remain to be determined. In any case, our analysis of the *nrpd1a-8* mutation has identified one more SI modifier locus that exhibits natural variation among *A. thaliana* accessions, similar to *PUB8*, which also regulates *SRK* transcript levels (Liu *et al.* 2007), and to a previously identified SI modifier on chromosome 3 (Boggs *et al.* 2009). The polymorphic nature of these loci supports the conclusion that the switch to self-fertility in *A. thaliana* was accompanied, not only by inactivation of *SRK* or *SCR* genes, but also by mutations at SI modifier loci that arose stochastically in different accessions.

## Supplementary Material

Supporting Information
