# Supplementary material for: Regulation of the S-Locus Receptor Kinase and Self-Incompatibility in Arabidopsis thaliana
Source: G3 (Bethesda). 2013 Feb 1;3(2):315–22. doi: 10.1534/g3.112.004879 (PMC3564991; doi:10.1534/g3.112.004879)
Supplement: Supporting Information [file supp_3_2_315__index.html]

Supporting Information 

# Regulation of the S-Locus Receptor Kinase and Self-Incompatibility in *Arabidopsis thaliana*

## Supporting Information for Strickler *et al.*, 2013

**Files in this Data Supplement:**

- Supporting Information - Figures S1-S2 and Tables S1-S4 (PDF, 387 KB)
- Figure S1 - RNA gel blot analysis of *SRKb* expression in *A. lyrata* tissues (PDF, 216 KB)
- Figure S2 - Loss of genome methylation in *rdr2*, *ago4*, and *nrpd1b* mutants as determined by chop-PCR analysis of the *AtSN1* retroelement (PDF, 225 KB)
- Table S1 - Primers used for mapping the *sc1* mutation (PDF, 222 KB)
- Table S2 - Analysis of a sample of *SRKb*-containing plants derived from the Col-0 *nrpd1a-8*[*SRKb*] x C24 WT[*SRKb-SCRb*] cross (PDF, 271 KB)
- Table S3 - Analysis of plants derived from the Col-0 *nrpd1a-8*[*SRKb*] x Sha WT[*SRKb-SCRb*] cross (PDF, 267 KB)
- Table S4 - Preliminary mapping of a putative modifier that segregates in F2 plants derived from the Col-0[*nrpd1a-8*] x C24[*SRKb-SCRb*] cross (PDF, 263 KB)
