# Supplementary material for: Regulation of the S-Locus Receptor Kinase and Self-Incompatibility in Arabidopsis thaliana
Source: G3 (Bethesda). 2013 Feb 1;3(2):315–22. doi: 10.1534/g3.112.004879 (PMC3564991; doi:10.1534/g3.112.004879)
Supplement: Supporting Information [file supp_3.2.315_004879SI.pdf]

G3: Genes | Genomes | Genetics

REGULATION of the S-LOCUS RECEPTOR KINASE and SELF-INCOMPATIBILITY in *ARABIDOPSIS THALIANA*

Susan R. Strickler, Titima Tantikanjana, and June B. Nasrallah

Department of Plant Biology, 412 Mann Library Building, Cornell University, Ithaca, NY 14853, USA

DOI: 10.1534/g3.112.004879

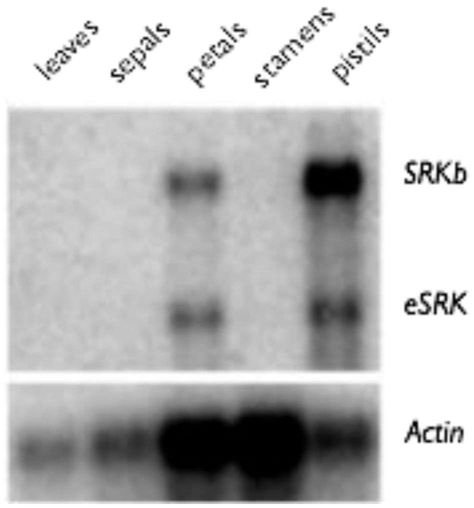

Figure S1 RNA gel blot analysis of *SRKb* expression in *A. lyrata* tissues. The blot was hybridized first with the *SRKb* probe (upper panel) and subsequently with an actin probe (lower panel).

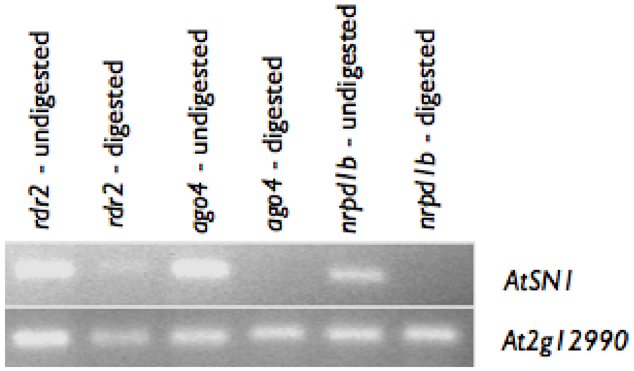

Figure S2 Loss of genome methylation in *rdr2*, *ago4*, and *nrpd1b* mutants as determined by chop-PCR analysis of the *AtSN1* retroelement.

Table S1   Primers used for mapping the *sc1* mutation.

Primer Name	Chrom	Primer 1 Sequence	Primer 2 Sequence
NGA63	1	ACCCAAGTGATCGCCACC	AACCAAGGCACAGAAGCG
CIW12	1	AGGTTTTATTGCTTTTCACA	CTTTCAAAAGCACATCACA
SO392	1	GTTGATCGCAGCTTGATAAGC	TTTGGAGTTAGACACGGATCTG
T27K12-SP6	1	GGAGGCTATACGAATCTTGACA	GGACAACGTCTCAAACGGTT
GAPB.2	1	CACTATGTTCAGTGCTGCG	GATCACTTGCAGCTATGGC
CIW1	1	ACATTTTCTCAATCCTTACTC	GAGAGCTTCTTTATTTGTGAT
NGA280	1	GGCTCCATAAAAAGTGCACC	CTGATCTCACGGACAATAGTGC
F8A5a	1	GCAGAGCATAAAGCCATAAACA	CGTGCATGTTGTTGGAATCT
T13M11b	1	AAGTCCACAAAGGAGGAGAAA	CCCTTCCTAAGCCTAGATTTTGT
F24O1a	1	TGCTACCTGTTGCAACCTCA	TTTGCTTTCGATGTGAAATGA
F23N19a	1	GGAAGGAGCCTGAGGTAGAGA	CAGCATTCCCCAACTCTTTC
F16P17a	1	TGAGACATTTCCTACATTATTTATTTG	AATTACATAAGTTTGATGTTTGTGCT
F16P17b	1	CTTTGGCTTGGTCACTAATATGTTCA	TTCAATGCTGGTTCACAGAAA
F16P17c	1	CCTGATGTTGTTTCATTTCGATTAG	GTGCTCTTGGAATGGCTTGT
F16M19a	1	TCAGTAATCAGAGAGAGAGATTTAGGG	AACTCGCCATTGACACAACA
F9N12c_col	1	GGACATTATGTTGAACCTCGTTT	TTCAACAACCTTTTGGTAAATAAGA
F9N12c_ler	1	GACATTATGTTGAACCTCGTGA	TTCAACAACCTTTTGGTAAATAAGA
F9N12b	1	ACTGAGGACAAATGTTTTGTAGC	TCGAAACAAAGCAGGAGGTT
F2K11b	1	TCCAAATGATGATCCTGCAA	CCGGCTTATGCAGTAAGAAA
F2K11a	1	TGGTCGTCACTCTCGTTCAG	CAGATGAGACAATTCAGGGAGA
F24D7a	1	TCACCCAATCTCCCCATAAG	CGAGCGCCTTACTCTGTGAT
F24D7b	1	AACCAGTTTTCAAATCAACTGAAG	TGGTGGTGTTGAGGTACCAA
T12P18b	1	CGTGCGCAGTTATCTCCTTT	TTATCACAGTTTTCATTTACCAAAAA
T12P18a	1	AGATAAATGCATCAACAAATTGAC	ACCCACCTCACACTCTCTCC
NGA111	1	TGTTTTTTAGGACAAATGGCG	CTCCAGTTGGAAGCTAAAGGG
NGA1145	2	GCACATACCCACAACCAGAA	CCTTCACATCCAAAACCCAC
CIW3	2	GAAACTCAATGAAATCCACTT	TGAACTTGTTGTGAGCTTTGA
NGA1126	2	GCACAGTCCAAGTCACAACC	CGCTACGCTTTTCGGTAAAG
NGA168	2	GAGGACATGTATAGGAGCCTCG	TCGTCTACTGCACTGCCG
NGA172	3	CATCCGAATGCCATTGTTC	AGCTGCTTCCTTATAGCGTCC
NGA162	3	CTCTGTCACTCTTTTCCTCTGG	CATGCAATTTGCATCTGAGG
CIW11	3	CCCCGAGTTGAGGTATT	GAAGAAATTCCTAAAGCATTC
T27C7-SP6	3	ATGCCTAACTATTCGCTGAC	TTCTGTAGTTCTTTGTGAGTGC
F1P2-TGF	3	TTTGTCTGAAGATGTGGAGAGAGAG	CAAAACCCCACTCTTCATTATTGTT
NGA6	3	ATGGAGAAGCTTACACTGATC	TGGATTTCTTCCTCTCTTCAC

CIW5	4	GGTTAAAAATTAGGGTTACGA	AGATTTACGTGGAAGCAAT
NGA8	4	TGGCTTTCGTTTATAAACATCC	GAGGGCAAATCTTTATTTCGG
CIW7	4	AATTTGGAGATTAGCTGGAAT	CCATGTTGATGATAAGCACAA
NGA1107	4	CGACGAATCGACAGAATTAGG	GCGAAAAAACAAAAAAATCCA
CTR1.2	5	CCACTTGTTTCTCTCTCTAG	TATCAACAGAAACGCACCGAG
CA72	5	CCCAGTCTAACCACGACCAC	AATCCCAGTAACCAAACACACA
NGA139	5	GGTTTCGTTTCACTATCCAGG	AGAGCTACCAGATCCGATGG
ATHPHYC	5	CTCAGAGAATTCCCAGAAAAATCT	AAACTCGAGAGTTTTGTCTAGATC
CIW9	5	CAGACGTATCAAATGACAAATG	GACTACTGCTCAAACTATTCGG
MTH12	5	GTAAAATTTTCTATTGCA	ATGTCCTCCTGTTCTGTCCA

Table S2 Analysis of a sample of *SRKb*-containing plants derived from the Col-0 *nrpd1a-8*[*SRKb*] x C24 WT[*SRKb-SCRb*] cross.

F2 Plant	Pollination Tests[a]		*nrpd1a* homozygote	*SRKb* Integration Number[b]	Genomic Methylation[c]
1-7	+++	+++	yes	1	-
1-8	+++	+++	yes	2	-
1-9	0	0	yes	1	-
1-11	0	0	yes	2	-
2-1	0	0	no	2	+
2-3	0	3	yes	1	-
2-4	0	0	no	1	+
2-6	0	0	yes	1	-
2-8	0	0	no	2	+
2-10	0	0	no	2	+
3-1	0	0	no	2	+
3-2	0	0	no	2	+
3-3	0	0	no	2	+
3-4	0	0	no	2	+
3-5	0	0	no	2	+
3-7	0	0	no	2	+
3-8	0	8	yes	1	-
3-9	0	0	no	2	+
3-11	3	0	yes	1	-
3-12	+	20	yes	1	-
6-3	+	++	yes	1	-
6-4	0	0	no	2	+
6-5	0	0	no	2	+
6-6	0	0	no	2	+
6-7	0	0	no	2	+
6-8	0	0	no	2	+
6-10	0	X	no	2	+
6-11	0	0	no	1	+

[a] Two replicate pollinations are shown. + indicates 20-50 pollen tubes per polinated stigma, ++ indicates 50-75 pollen tubes per pollinated stigma, +++ indicates over 75 pollen tubes per pollinated stigma.
[b] The number of *SRKb* transgene integrations
[c] Determined by *AtSN1* methylation

Table S3 Analysis of plants derived from the Col-0 *nrpd1a-8*[*SRKb*] x Sha WT[*SRKb-SCRb*] cross. All selected plants had the *SRKb* transgene.

F2 Plant	Pollination Tests[a]		*nrpd1a* homozygote
1-1	0	0	yes
1-2	3	0	no
2-1	0	2	no
2-2	0	0	no
2-3	0	0	no
2-4	0	0	no
2-5	0	3	yes
2-6	0	2	no
2-9	0	0	no
2-10	2	0	no
2-12	0	0	no
2-13	++	+	yes
3-1	0	0	yes
3-2	1	1	yes
3-5	0	0	no
3-6	0	0	no
3-8	0	0	yes
3-9	1	0	no
3-11	0	0	yes
3-12	0	0	no
4-3	0	0	no
4-5	0	0	no
4-7	0	0	no
4-8	25	20	yes
4-11	0	0	yes
4-12	0	0	no

[a] Two replicate pollinations are shown. + indicates 20-50 pollen tubes per pollinated stigma, ++ indicates 50-75 pollen tubes per pollinated stigma, +++ indicates over 75 pollen tubes per pollinated stigma.

Table S4 Preliminary mapping of a putative modifier that segregates in F2 plants derived from the Col-0[*nrpd1a-8*] x C24[*SRKb-SCRb*] cross. All plants listed are *nrpd1a-8 SRKb* homozygotes.

F2 Family	Pollination Tests[a]		NGA139[b]	CIW9[c]
1-7	+++	+++	H	H
1-8	+++	+++	Col	Col
3-12	+	20	Col	H
6-3	+	++	Col	Col
1-1	0	1	H	H
2-3	0	3	C24	-
2-6	0	0	H	H
3-11	3	0	H	Col

[a] + indicates 20-50 pollen tubes observed, ++ indicates 50-75 pollen tubes observed, +++ indicates over 75 pollen tubes
[b] marker located at 8.5 megabases on chromosome 5
[c] marker located at 17.1 megabases on chromosome 5
