# Supplementary material for: Regulation of the S-Locus Receptor Kinase and Self-Incompatibility in Arabidopsis thaliana
Source: G3 (Bethesda). 2013 Feb 1;3(2):315–22. doi: 10.1534/g3.112.004879 (PMC3564991; doi:10.1534/g3.112.004879)
Supplement: Supporting Information [file supp_3.2.315_FigureS1.pdf]

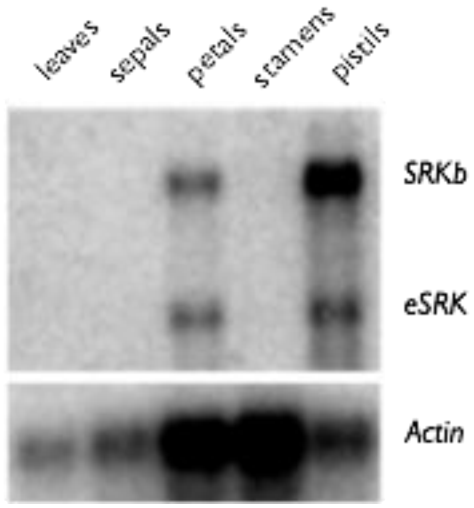

Figure S1 RNA gel blot analysis of *SRKb* expression in *A. lyrata* tissues. The blot was hybridized first with the *SRKb* probe (upper panel) and subsequently with an actin probe (lower panel).
