# Supplementary material for: Regulation of the S-Locus Receptor Kinase and Self-Incompatibility in Arabidopsis thaliana
Source: G3 (Bethesda). 2013 Feb 1;3(2):315–22. doi: 10.1534/g3.112.004879 (PMC3564991; doi:10.1534/g3.112.004879)
Supplement: Supporting Information [file supp_3.2.315_FigureS2.pdf]

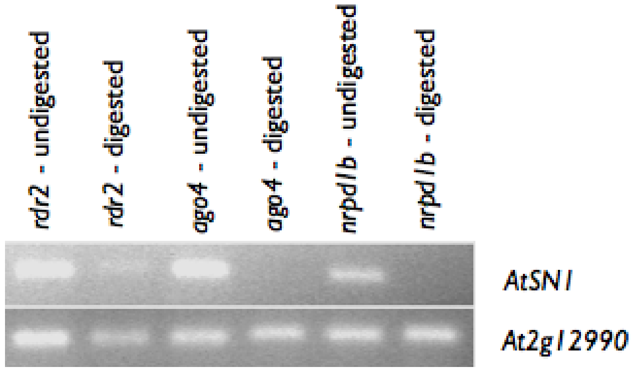

Figure S2 Loss of genome methylation in *rdr2*, *ago4*, and *nrpd1b* mutants as determined by chop-PCR analysis of the *AtSN1* retroelement.
