# Supplementary material for: Regulation of the S-Locus Receptor Kinase and Self-Incompatibility in Arabidopsis thaliana
Source: G3 (Bethesda). 2013 Feb 1;3(2):315–22. doi: 10.1534/g3.112.004879 (PMC3564991; doi:10.1534/g3.112.004879)
Supplement: Supporting Information [file supp_3.2.315_TableS1.pdf]

Table S1 Primers used for mapping the *sc1* mutation.

Primer Name	Chrom	Primer 1 Sequence	Primer 2 Sequence
NGA63	1	ACCCAAGTGATCGCCACC	AACCAAGGCACAGAAGCG
CIW12	1	AGGTTTTATTGCTTTTCACA	CTTTCAAAAGCACATCACA
SO392	1	GTTGATCGCAGCTTGATAAGC	TTTGGAGTTAGACACGGATCTG
T27K12-SP6	1	GGAGGCTATACGAATCTTGACA	GGACAACGTCTCAAACGGTT
GAPB.2	1	CACTATGTTCAGTGCTGCG	GATCACTTGCAGCTATGGC
CIW1	1	ACATTTTCTCAATCCTTACTC	GAGAGCTTCTTTATTTGTGAT
NGA280	1	GGCTCCATAAAAAGTGCACC	CTGATCTCACGGACAATAGTGC
F8A5a	1	GCAGAGCATAAAGCCATAAACA	CGTGCATGTTGTTGGAATCT
T13M11b	1	AAGTCCACAAAGGAGGAGAAA	CCCTTCCTAAGCCTAGATTTTGT
F24O1a	1	TGCTACCTGTTGCAACCTCA	TTTGCTTTCGATGTGAAATGA
F23N19a	1	GGAAGGAGCCTGAGGTAGAGA	CAGCATTCCCCAACTCTTTC
F16P17a	1	TGAGACATTTCCTACATTATTTATTTG	AATTACATAAGTTTGATGTTTGTGCT
F16P17b	1	CTTTGGCTTGGTCACTAATATGTTCA	TTCAATGCTGGTTCACAGAAA
F16P17c	1	CCTGATGTTGTTTCATTTCGATTAG	GTGCTCTTGGAATGGCTTGT
F16M19a	1	TCAGTAATCAGAGAGAGAGATTTAGGG	AACTCGCCATTGACACAACA
F9N12c_col	1	GGACATTATGTTGAACCTCGTTT	TTCAACAACCTTTTGGTAAATAAGA
F9N12c_ler	1	GACATTATGTTGAACCTCGTGA	TTCAACAACCTTTTGGTAAATAAGA
F9N12b	1	ACTGAGGACAAATGTTTTGTAGC	TCGAAACAAAGCAGGAGGTT
F2K11b	1	TCCAAATGATGATCCTGCAA	CCGGCTTATGCAGTAAGAAA
F2K11a	1	TGGTCGTCACTCTCGTTCAG	CAGATGAGACAATTCAGGGAGA
F24D7a	1	TCACCCAATCTCCCCATAAG	CGAGCGCCTTACTCTGTGAT
F24D7b	1	AACCAGTTTTCAAATCAACTGAAG	TGGTGGTGTTGAGGTACCAA
T12P18b	1	CGTGCGCAGTTATCTCCTTT	TTATCACAGTTTTCATTTACCAAAAA
T12P18a	1	AGATAAATGCATCAACAAATTGAC	ACCCACCTCACACTCTCTCC
NGA111	1	TGTTTTTTAGGACAAATGGCG	CTCCAGTTGGAAGCTAAAGGG
NGA1145	2	GCACATACCCACAACCAGAA	CCTTCACATCCAAAACCCAC
CIW3	2	GAAACTCAATGAAATCCACTT	TGAACTTGTTGTGAGCTTTGA
NGA1126	2	GCACAGTCCAAGTCACAACC	CGCTACGCTTTTCGGTAAAG
NGA168	2	GAGGACATGTATAGGAGCCTCG	TCGTCTACTGCACTGCCG
NGA172	3	CATCCGAATGCCATTGTTC	AGCTGCTTCCTTATAGCGTCC
NGA162	3	CTCTGTCACTCTTTTCCTCTGG	CATGCAATTTGCATCTGAGG
CIW11	3	CCCCGAGTTGAGGTATT	GAAGAAATTCCTAAAGCATTC
T27C7-SP6	3	ATGCCTAACTATTCGCTGAC	TTCTGTAGTTCTTTGTGAGTGC
F1P2-TGF	3	TTTGTCTGAAGATGTGGAGAGAGAG	CAAAACCCCACTCTTCATTATTGTT
NGA6	3	ATGGAGAAGCTTACACTGATC	TGGATTTCTTCCTCTCTTCAC

CIW5	4	GGTTAAAAATTAGGGTTACGA	AGATTTACGTGGAAGCAAT
NGA8	4	TGGCTTTCGTTTATAAACATCC	GAGGGCAAATCTTTATTTCGG
CIW7	4	AATTTGGAGATTAGCTGGAAT	CCATGTTGATGATAAGCACAA
NGA1107	4	CGACGAATCGACAGAATTAGG	GCGAAAAAACAAAAAAATCCA
CTR1.2	5	CCACTTGTTTCTCTCTCTAG	TATCAACAGAAACGCACCGAG
CA72	5	CCCAGTCTAACCACGACCAC	AATCCCAGTAACCAAACACACA
NGA139	5	GGTTTCGTTTCACTATCCAGG	AGAGCTACCAGATCCGATGG
ATHPHYC	5	CTCAGAGAATTCCCAGAAAAATCT	AAACTCGAGAGTTTTGTCTAGATC
CIW9	5	CAGACGTATCAAATGACAAATG	GACTACTGCTCAAACTATTCGG
MTH12	5	GTAAAATTTTCTATTGCA	ATGTCCTCCTGTTCTGTCCA
