# Supplementary material for: Regulation of the S-Locus Receptor Kinase and Self-Incompatibility in Arabidopsis thaliana
Source: G3 (Bethesda). 2013 Feb 1;3(2):315–22. doi: 10.1534/g3.112.004879 (PMC3564991; doi:10.1534/g3.112.004879)
Supplement: Supporting Information [file supp_3.2.315_TableS2.pdf]

Table S2 Analysis of a sample of *SRKb*-containing plants derived from the Col-0 *nrpd1a-8*[*SRKb*] x C24 WT[*SRKb*-*SCRb*] cross.

F2 Plant	Pollination Tests[a]		*nrpd1a* homozygote	*SRKb* Integration Number[b]	Genomic Methylation[c]
1-7	+++	+++	yes	1	-
1-8	+++	+++	yes	2	-
1-9	0	0	yes	1	-
1-11	0	0	yes	2	-
2-1	0	0	no	2	+
2-3	0	3	yes	1	-
2-4	0	0	no	1	+
2-6	0	0	yes	1	-
2-8	0	0	no	2	+
2-10	0	0	no	2	+
3-1	0	0	no	2	+
3-2	0	0	no	2	+
3-3	0	0	no	2	+
3-4	0	0	no	2	+
3-5	0	0	no	2	+
3-7	0	0	no	2	+
3-8	0	8	yes	1	-
3-9	0	0	no	2	+
3-11	3	0	yes	1	-
3-12	+	20	yes	1	-
6-3	+	++	yes	1	-
6-4	0	0	no	2	+
6-5	0	0	no	2	+
6-6	0	0	no	2	+
6-7	0	0	no	2	+
6-8	0	0	no	2	+
6-10	0	X	no	2	+
6-11	0	0	no	1	+

[a] Two replicate pollinations are shown. + indicates 20-50 pollen tubes per polinated stigma, ++ indicates 50-75 pollen tubes per pollinated stigma, +++ indicates over 75 pollen tubes per pollinated stigma.
[b] The number of *SRKb* transgene integrations
[c] Determined by *AtSN1* methylation
