# Supplementary material for: Regulation of the S-Locus Receptor Kinase and Self-Incompatibility in Arabidopsis thaliana
Source: G3 (Bethesda). 2013 Feb 1;3(2):315–22. doi: 10.1534/g3.112.004879 (PMC3564991; doi:10.1534/g3.112.004879)
Supplement: Supporting Information [file supp_3.2.315_TableS3.pdf]

Table S3 Analysis of plants derived from the Col-0 *nrpd1a-8*[*SRKb*] x Sha WT[*SRKb-SCRb*] cross. All selected plants had the *SRKb* transgene.

F2 Plant	Pollination Tests[a]		*nrpd1a* homozygote
1-1	0	0	yes
1-2	3	0	no
2-1	0	2	no
2-2	0	0	no
2-3	0	0	no
2-4	0	0	no
2-5	0	3	yes
2-6	0	2	no
2-9	0	0	no
2-10	2	0	no
2-12	0	0	no
2-13	++	+	yes
3-1	0	0	yes
3-2	1	1	yes
3-5	0	0	no
3-6	0	0	no
3-8	0	0	yes
3-9	1	0	no
3-11	0	0	yes
3-12	0	0	no
4-3	0	0	no
4-5	0	0	no
4-7	0	0	no
4-8	25	20	yes
4-11	0	0	yes
4-12	0	0	no

[a] Two replicate pollinations are shown. + indicates 20-50 pollen tubes per pollinated stigma, ++ indicates 50-75 pollen tubes per pollinated stigma, +++ indicates over 75 pollen tubes per pollinated stigma.
