# Supplementary material for: Regulation of the S-Locus Receptor Kinase and Self-Incompatibility in Arabidopsis thaliana
Source: G3 (Bethesda). 2013 Feb 1;3(2):315–22. doi: 10.1534/g3.112.004879 (PMC3564991; doi:10.1534/g3.112.004879)
Supplement: Supporting Information [file supp_3.2.315_TableS4.pdf]

Table S4 Preliminary mapping of a putative modifier that segregates in F2 plants derived from the Col-0[*nrpd1a-8*] x C24[*SRKb-SCRb*] cross. All plants listed are *nrpd1a-8 SRKb* homozygotes.

F2 Family	Pollination Tests[a]		NGA139[b]	CIW9[c]
1-7	+++	+++	H	H
1-8	+++	+++	Col	Col
3-12	+	20	Col	H
6-3	+	++	Col	Col
1-1	0	1	H	H
2-3	0	3	C24	-
2-6	0	0	H	H
3-11	3	0	H	Col

[a] + indicates 20-50 pollen tubes observed, ++ indicates 50-75 pollen tubes observed, +++ indicates over 75 pollen tubes

[b] marker located at 8.5 megabases on chromosome 5

[c] marker located at 17.1 megabases on chromosome 5
